# Phytochemical Analysis and In Vitro Cytotoxic Activity against Colorectal Adenocarcinoma Cells of *Hippophae rhamnodies* L., *Cymbopogon citratus* (D.C.) Stapf, and *Ocimum basilicum* L. Essential Oils

**DOI:** 10.3390/plants10122752

**Published:** 2021-12-14

**Authors:** Alina Dolghi, Roxana Buzatu, Amadeus Dobrescu, Flavius Olaru, Grigore Alexandru Popescu, Iasmina Marcovici, Iulia Pinzaru, Dan Navolan, Octavian Marius Cretu, Iuliana Popescu, Cristina Adriana Dehelean

**Affiliations:** 1 Department of Toxicology and Drug Industry, Faculty of Pharmacy, “Victor Babes” University of Medicine and Pharmacy Timisoara, Eftimie Murgu Sq. No. 2, 300041 Timisoara, Romania; dolghi.alina@umft.ro (A.D.); iasmina.marcovici@umft.ro (I.M.); iuliapinzaru@umft.ro (I.P.); cadehelean@umft.ro (C.A.D.); 2 Research Center for Pharmaco-Toxicological Evaluations, “Victor Babes” University of Medicine and Pharmacy Timisoara, Eftimie Murgu Sq. No. 2, 300041 Timisoara, Romania; 3 Department of Dental Aesthetics, Faculty of Dental Medicine, “Victor Babeș” University of Medicine and Pharmacy Timisoara, Eftimie Murgu Sq. No. 2, 300041 Timișoara, Romania; drbuzaturoxana@gmail.com; 4 Department of Surgery, Faculty of Medicine, “Victor Babes” University of Medicine and Pharmacy Timisoara, Eftimie Murgu Sq. No. 2, 300041 Timisoara, Romania; grigore.popescu@umft.ro (G.A.P.); octavian.cretu@umft.ro (O.M.C.); 5 Department of Obstetrics-Gynecology, Faculty of Medicine, “Victor Babes” University of Medicine and Pharmacy Timisoara, Eftimie Murgu Sq. No. 2, 300041 Timisoara, Romania; navolan@umft.ro; 6 Department of Soil Sciences, Faculty of Agriculture, Banat University of Agricultural Sciences and Veterinary Medicine “King Michael I of Romania”, Calea Aradului 119, 300645 Timişoara, Romania; iuliana_popescu@usab-tm.ro

**Keywords:** essential oils, tumor cells, viability, nuclear morphology, migratory capacity

## Abstract

Colorectal carcinoma (CRC) is one of the most frequently diagnosed cancer types with current deficient and aggressive treatment options, but various studied alternative therapies are able to efficiently contribute to its management. Essential oils (EOs) contain valuable compounds, with antibacterial, anti-inflammatory, and anticancer properties, which might serve as effective solutions in CRC prophylaxis or treatment. The aim of the present work was to evaluate the phytochemical composition and in vitro biological activity of essential oils derived from *Hippophae rhamnoides* (Hr_EO), *Cymbopogon citratus* (Cc_EO), and *Ocimum basilicum* (Ob_EO) species on HT-29 and Caco-2 human colorectal adenocarcinoma cell lines. The main compounds identified by GC-MS analysis were estragole (Hr_EO, Ob_EO), alpha- and beta-citral (Cc_EO). All tested EOs exerted a dose-dependent cytotoxicity on both cell lines by reducing the cell viability, especially in the case of Cc_EO, where at 75 µg/mL the viability percentages reached the values of 62.69% (Caco-2) and 64.09% (HT-29), respectively. The nuclear morphology evaluation highlighted significant dysmorphologies on both lines after their treatment with EOs at 75 µg/mL.

## 1. Introduction

Colorectal carcinoma (CRC) represents the fourth most frequently diagnosed malignancy, and the third leading cause of cancer-related deaths in adults worldwide. According to the most recent GLOBOCAN data, the estimated CRC incidence is 19.5/100,000 inhabitants, and following an ascending trend line [1]. Although the pathogenetic mechanisms leading to CRC are not fully understood [2], several risk factors have been strongly correlated to its onset, such as age, gene mutations (i.e., APC, KRAS, TP53), pre-existent digestive pathologies (e.g., inflammatory bowel disease, Crohn’s disease), lifestyle habits (e.g, diet, sedentarism, smoking, alcohol consumption), and dysbiosis [3]. 

Curative treatment regimens vary from surgical resection in incipient stages to neoadjuvant therapy in advanced CRC forms, referring to radiotherapy and chemotherapy medication (e.g., leucovorin calcium, 5-Fluorouracil, oxyplatine, fluoropyrimidine, capecitabine) [4]. Although the current chemotherapy significantly have been shown to increase patients’ survival rate, improve the CRC outcome, and reduce the disease recurrence to a considerable extent, severe adverse events and drug-resistance are important disadvantages limiting this treatment option [5]. Therefore, the development of alternative therapy approaches is required. Medicinal plants play a substantial role in the prophylaxis and treatment of various types of cancer [6], and stand as an inexhaustible resource for phytochemicals possessing anti-CRC properties due to their antioxidant, anti-inflammatory, pro-apoptotic, and proautophagic effects [7]. Essential oils (EOs) are volatile, aromatic, hydrophobic, plant-derived products traditionally used in therapy for their broad spectrum of biological activities (e.g., sedative, antiseptic, anti-inflammatory, antimicrobial, spasmolytic, and anti-cancer) [8], which might offer substantial benefits in the management of colonic pathology [9]. 

*Hippophae rhamnoides* L. (sea buckthorn) is a medicinal plant widely recognized for exerting anti-inflammatory, antioxidant, anti-atherosclerotic, and anti-cancer activities due to the abundance of bioactive compounds found in its berries, leaves, roots, seeds, and oil. The anti-tumor property is mainly attributed to antioxidant compounds, such as flavonoids which offer protection against oxidative stress-induced damage and genetic mutations [10]. The composition of sea buckthorn essential oils is dominated by free fatty acids, esters, and alkanes, which print an important biological potency [11]. Several studies suggest a broad anticancer spectrum on lung, liver, breast, and blood malignancies [12,13,14,15].

*Cymbopogon citratus* (D.C.) Stapf or lemongrass is an aromatic herb widely exploited for its essential oil, which possesses a plethora of pharmacological effects varying from anti-amebic, anti-fungal, anti-malarial and anti-bacterial to anti-oxidant and anti-inflammatory properties [16]. Recent publications refer to the anti-tumor activity of lemongrass extracts against prostate, liver, ovarian, colon, and breast carcinomas [17,18]. 

*Ocimum basilicum* L. or basil is a plant belonging to the Lamiaceae family. Beside its traditional use for culinary purposes, basil exerts several biological activities (e.g., anti-microbial, anti-diabetic, cardio-protective, anti-inflammatory, anti-cancer, and chemopreventive) providing multiple health benefits [19].

The aim of the present work was to evaluate the phytochemical composition and investigate the in vitro anti-tumor capacity of *H. rhamnoides* L., *C. citratus* (D.C.) Stapf, and *O. basilicum* L. essential oils (Hr_EO, Cc_EO, and Ob_EO) as potential chemo-prophylactic or chemo-therapeutic alternatives in CRC management. To the best of our knowledge, this is the first study in the literature that investigates the anti-CRC properties of these essential oils against two human colorectal adenocarcinoma cells (Caco-2 and HT-29).

## 2. Results

### 2.1. Gas Chromatography-Mass Spectrometry (GC-MS) Analysis

In order to evaluate the phytochemical composition of commercial Hr_EO, Cc_EO, and Oc_EO, a GC-MS analysis was performed. Therefore, in Hr_EO there were 13 compounds identified (presented in Table 1), the most abundant being estragole representing 63.1% of the total oil composition. In Cc_EO, the most abundant compounds among the 33 detected (displayed in Table 2) were alpha- and beta-citral, accounting for 66.2% of the total oil composition. Similarly to Hr_EO, in Ob_EO, estragole was the main abundant compound among the 31 identified, with a total percentage of 45.9 %. The complete composition is displayed in Table 3. 

### 2.2. Cell Viability Assessment

**Figure 1:**
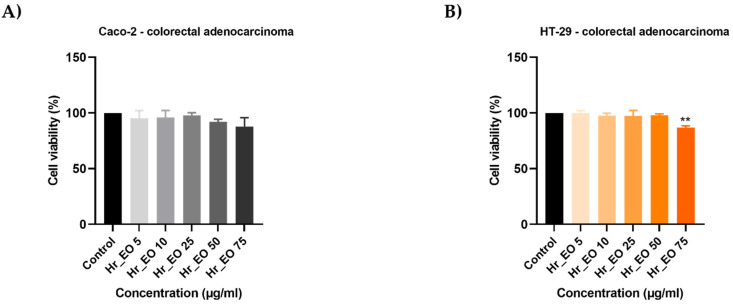
In vitro assessment of the effect Hr_EO (5, 10, 25, 50, and 75 µg/mL) exerts on the viability of (**A**) Caco-2 and (**B**) HT-29 colorectal adenocarcinoma cells after 48 h of treatment by applying the MTT assay. The data are presented as viability percentages (%) normalized to control (untreated cells) and expressed as mean values ± SD of three independent experiments performed in triplicate. The statistical differences between the control and the treated group were identified by applying the one-way ANOVA analysis followed by the Dunett’s multiple comparisons post-test (** *p* < 0.01).

**Figure 2:**
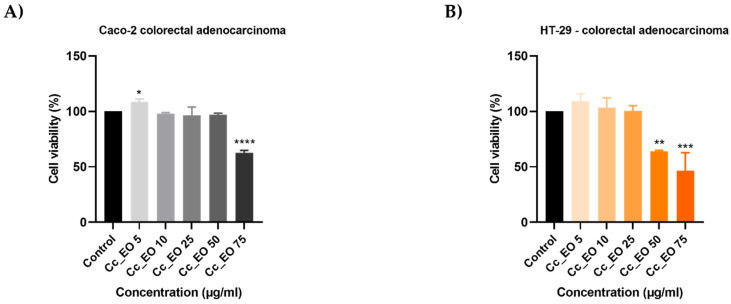
In vitro assessment of the effect Cc_EO (5, 10, 25, 50, and 75 µg/mL) exerts on the viability of (**A**) Caco-2 and (**B**) HT-29 colorectal adenocarcinoma cells after 48 h of treatment by applying the MTT assay. The data are presented as viability percentages (%) normalized to control (untreated cells) and expressed as mean values ± SD of three independent experiments performed in triplicate. The statistical differences between the control and the treated group were identified by applying the one-way ANOVA analysis followed by the Dunett’s multiple comparisons post-test (* *p* < 0.1; ** *p* < 0.01; *** *p* < 0.001; **** *p* < 0.0001).

**Figure 3:**
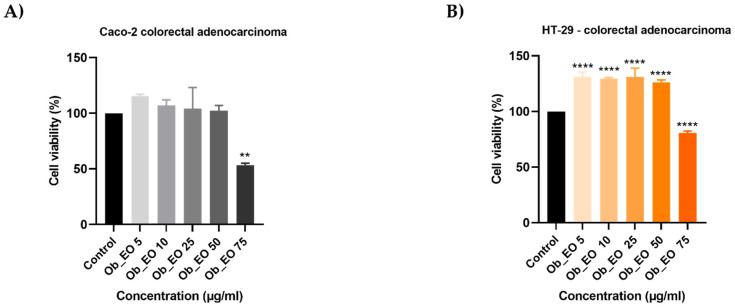
In vitro assessment of the effect Ob_EO (5, 10, 25, 50, and 75 µg/mL) exerts on the viability of (**A**) Caco-2 and (**B**) HT-29 colorectal adenocarcinoma cells after 48 h of treatment by applying the MTT assay. The data are presented as viability percentages (%) normalized to control (untreated cells) and expressed as mean values ± SD of three independent experiments performed in triplicate. The statistical differences between the control and the treated group were identified by applying the one-way ANOVA analysis followed by the Dunett’s multiple comparisons post-test (** *p* < 0.01; **** *p* < 0.0001).

In order to analyze the capacity of EOs to inhibit cell proliferation, the 3-(4,5)-dimethylthiazol-2-yl-2,5-diphenyltetrazolium bromide (MTT) assay was performed. Five concentrations (5, 10, 25, 50, 75 µg/mL) of each oil considered were tested on HT-29 and Caco-2 human adenocarcinoma cell lines for 48 h. In all cases, the viability percentages varied in a concentration-dependent manner, EOs displaying an anti-cancer effect only at high concentrations. Hr_EO (Figure 1) exerts a similar cytotoxic activity in both cell lines, the most significant effect being registered at 75 µg/mL when the viability percentages reached the values of 87.83% (Caco-2) and 86.91% (HT-29). Cc_EO decreased the viability of Caco-2 cells only at 75 µg/mL (62.69%) (Figure 2A), while the viability of HT-29 cells declined after the 48 h treatment with Cc_EO 50 µg/mL (64.09%) and 75 µg/mL (46.58%) (Figure 2B). Similar to Hr_EO, Ob_EO significantly reduced the cell viability at the highest concentration tested (75 µg/mL–53.36% in Caco-2 cells (Figure 3A) and 80.67% in HT-29 cells (Figure 3B)), while at lower concentrations a stimulatory effect was noticed (all viability percentages were over 100%).

### 2.3. Cell Morphology and Confluence

**Figure 4:**
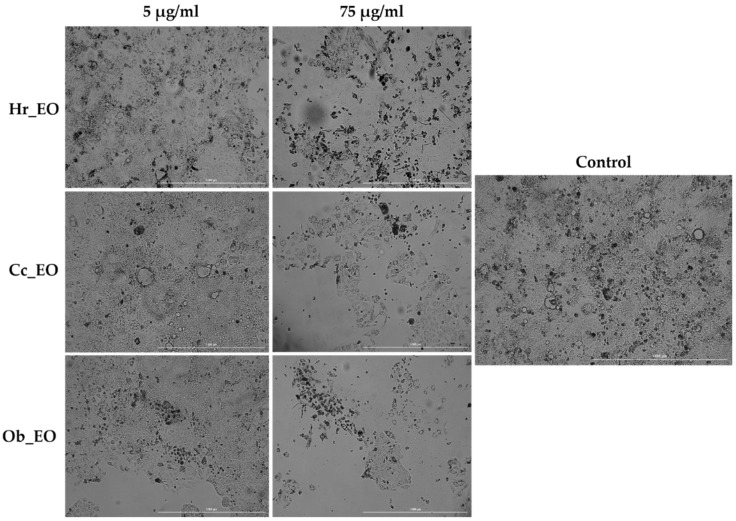
Pictures illustrating the morphological aspect and confluence of Caco-2 colorectal adenocarcinoma cells following the 48 h treatment with Hr_EO, Cc_EO, and Ob_EO (5 and 75 µg/mL). The scale bars represent 1000 µm.

As a component of the anti-cancer profile of EOs, a microscopic examination of the Caco-2 cells (Figure 4) was performed at the end of the 48 h treatment. The lowest (5 µg/mL) and the highest (75 µg/mL) concentrations were selected for this experiment. All three EOs (Hr_EO, Cc_EO, Ob_EO) induced a significant loss in the cells’ confluence and adherence at 75 µg/mL, while at 5 µg/mL no changes can be noted when compared to control.

### 2.4. Nuclear Morphology Evaluation

**Figure 5:**
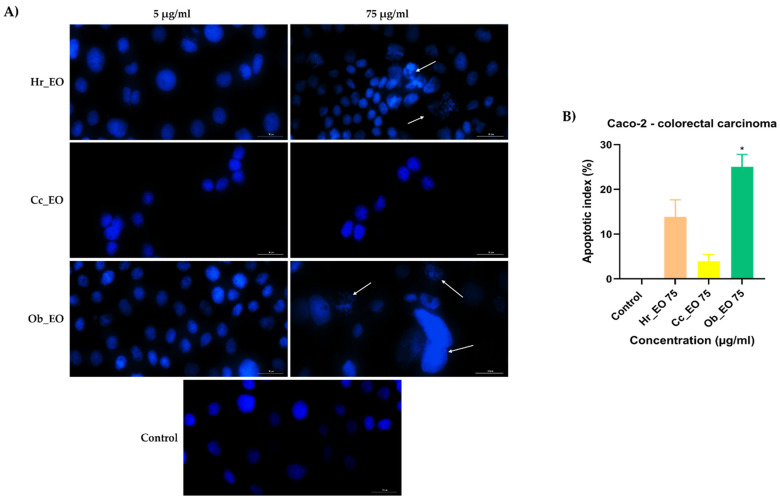
(**A**) Images of the cellular nuclei stained using Hoechst 33342 reagent in Caco-2 CRC cells following the 48 h treatment with Hr_EO, Cc_EO, and Ob_EO (5 and 75 µg/mL) and (**B**) calculated apoptotic index (AI) percentages for the highest concentration tested (75 µg/mL). The arrows indicate nuclei expressing apoptotic features. The scale bars represent 30 µm. Data are presented as an apoptotic index (%) normalized to control and expressed as mean values ± SD of three independent experiments. The statistical differences between control and the treated group were verified by applying the one-way ANOVA analysis followed by Dunnett’s multiple comparisons post-test (* *p* < 0.1).

**Figure 6:**
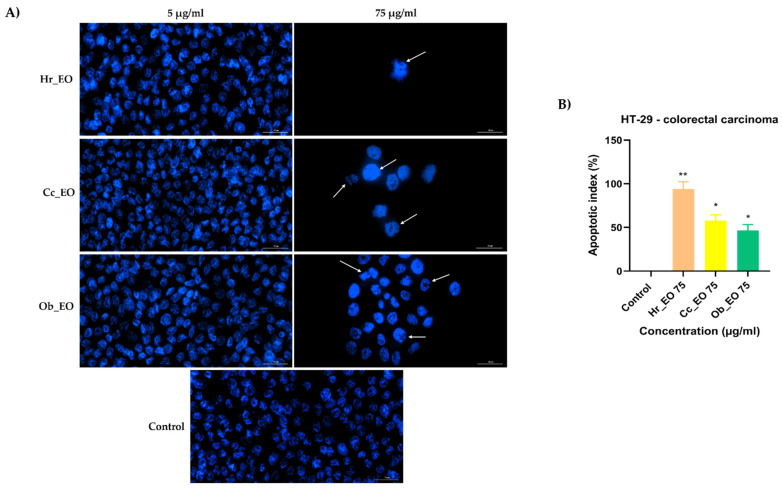
(**A**) Images of the cellular nuclei stained using Hoechst 33342 reagent in HT-29 CRC cells following the 48 h treatment with Hr_EO, Cc_EO, and Ob_EO (5 and 75 µg/mL) and (**B**) calculated apoptotic index (AI) percentages for the highest concentration tested (75 µg/mL). The arrows indicate nuclei expressing apoptotic features. The scale bars represent 30 µm. Data are presented as an apoptotic index (%) normalized to control and expressed as mean values ± SD of three independent experiments. The statistical differences between control and the treated group were verified by applying the one-way ANOVA analysis followed by Dunnett’s multiple comparisons post-test (* *p* < 0.1; ** *p* < 0.01).

Considering the viability results, the next step was a preliminary experiment to identify whether cell death occurred by apoptosis or necrosis. Hence, HT-29 and Caco-2 cells were stimulated for 48 h with two different concentrations (5 and 75 µg/mL) of Hr_EO, Cc_EO, and Ob_EO, the cell nuclei were counterstained using the Hoechst 33342 reagent, and the results were compared with control (unstimulated) cells. In the case of Caco-2 (Figure 5) and HT-29 (Figure 6) control cells, the nuclei have a round and regular shape, evenly stained, without signs of fragmentation. However, following the 48 h treatment with EOs, several changes in the aspect of the cellular nuclei were observed only at the highest concentration tested—75 µg/mL. Hr_EO induced nuclear fragmentation in Caco-2 cells, and membrane blebbing in HT-29 cells. Cc_EO induced visible dysmorphology only in HT-29 cells (nuclear condensation and fragmentation). In Caco-2 cells, Ob_EO caused nuclear fragmentation, chromatin condensation, and massive nuclear growth, while in HT-29 cells chromatin condensation and nuclei fragmentation were noticed. The results regarding the apoptotic-like features, are indicated by arrows. The Hoechst 33342 results are expressed as apoptotic index (AI) as well. All EOs induced an increase in the AI percentage as compared to control where no signs of apoptosis were detected. The most significant results were obtained in HT-29 cells at the concentration of 75 µg/mL with AI values of 94.27% (Hr_EO), 57.85% (Cc_EO), and 46.66% (Ob_EO). In Caco-2 cells, the registered AI percentages were 13.83% (Hr_EO), 3.93% (Cc_EO), 25.03% (Ob_EO) at 75 µg/mL.

### 2.5. Wound Healing Assay

**Figure 7:**
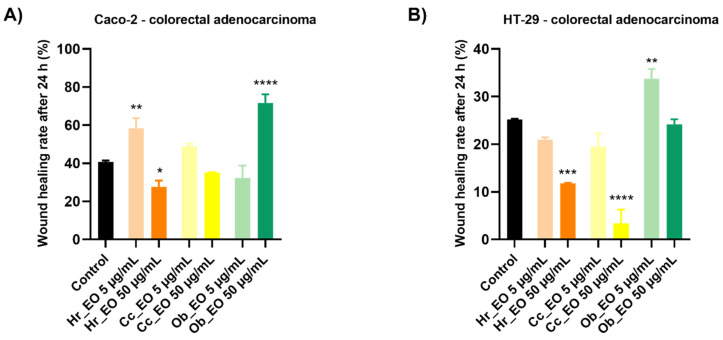
Graphic representation of the migratory capacity of Caco-2 (**A**) and HT-29 (**B**) colorectal adenocarcinoma cells following the treatment with Hr_EO, Cc_EO, and Ob_EO 5 and 50 µg/mL for 24 h. The bar graphs are presented as percentage of wound closure after 24 h compared to the initial surface. The data are expressed as mean values ± SD of three independent experiments performed in triplicate. To identify the statistical differences between the control and the treated group, the one-way ANOVA analysis was conducted followed by the Dunett’s multiple comparisons post-test (* *p* < 0.1; ** *p* < 0.001; *** *p* < 0.0001; **** *p* < 0.0001).

In order to evaluate the impact of EOs on the migration of Caco-2 and HT-29 cells, a wound healing assay was performed. The cells were treated with two concentrations (5 and 50 µg/mL) of each oil for 24 h. The results were highly depended on the tested oil, cell line, and concentration (Figure 7). Significant inhibition in the cells’ migration was noticed following their treatment with Hr_EO 50 µg/mL with wound healing rates of 27.61% (Caco-2) and 11.78% (HT-29), which are lower when compared to control (40.73%—Caco-2; 25.20%—HT-29). The same tendency was observed in the case of Cc_EO, but at 50 µg/mL it presented a much better capacity for migratory inhibition, especially on HT-29 cells, with a wound healing rate (WHR) of 3.40%. Ob_EO was generally associated with a significant increase in the cells’ migration rate.

### 2.6. HET-CAM Assay

**Figure 8:**
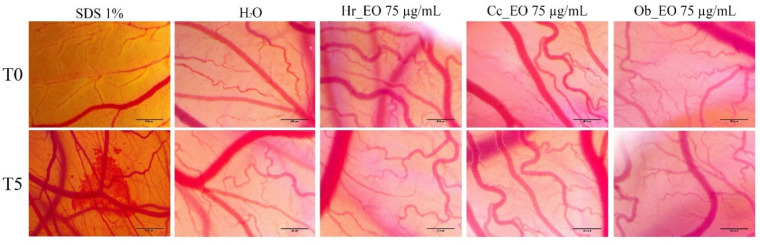
Stereomicroscope images of the CAMs inoculated with negative control (distilled water—H_2_O), positive control (sodium dodecyl sulfate—SDS), and essential oil test samples (Hr_EO, Cc_EO, and Ob_EO).

To determine the irritant potential of the three volatile oils (Hr_EO, Cc_EO, and Ob_EO), the HET-CAM method was applied considering that the safety profile is important for further in vivo evaluations. During the experiments, important data regarding the toxic potential of substances are provided both by calculating the irritation score and by analyzing the impact on vessels. To have a clear picture of the effects exerted by volatile oils, distilled water was used as negative control, while sodium dodecyl sulfate (SDS) 1% was a positive control. Thus, Table 4 presents the values of the irritation score obtained for the three samples, but also for the controls. In the case of positive control, SDS, the highest value of the irritation score of about 20 was obtained. At the opposite pole is distilled water, with a value of the irritation score being 0.1. Between these values are the three tested samples, the value of the irritation score in this case being closer to that of water, which suggests that all the essential oils tested were free of irritating effects on the vascular plexus. However, in Figure 8, the images made at the chorioallantoic membrane before and after the application of the substances can be observed. In the case of SDS, in the first minute after its application, strong irritating effects, such as lysis, coagulation, and vascular hemorrhage were registered at the level of the chorioallantoic membrane. In the case of volatile oils, they did not cause major changes in the vascular plexus, the only effect recorded being a slight intravascular coagulation, recorded at the end of the five minutes. However, the viability of chicken embryos was good, and they survived even after 24 h of application of the samples (Figure 8).

## 3. Discussion

Colorectal cancer remains one of the leading causes of death worldwide, with an incidence which is alarmingly growing, and with limited therapy options in advanced stages. Considering the multitude of natural sources available for compound isolation and their ability to prevent tumor occurrence, as well as target tumor cells after disease onset, plant-derived products have gained substantial interest in the area of cancer research [20]. To date, several publications direct the use of phytocompounds (e.g., flavonoids, stilbenes, terpenes) and plant extracts (e.g., *S. libanotica*, *H. rhamnodies*, *M. fragrance*, *C. sinensis*, *C.n citratus*, *M. piperita*, *O. basilicum* L.) toward CRC chemotherapy [15,21,22].

The purpose of the present study was to evaluate the quality of three commercial essential oils (Hr_EO, Ob_EO, and Cc_EO) in terms of phytochemical composition and in vitro anti-cancer effect on two CRC cell lines—Caco-2 and HT-29—which are isolated from human colon adenocarcinomas, and largely used as in vitro models in CRC studies. The Caco-2 cell line is capable to form polarized monolayers in culture and differentiates into cells with high homology to enterocytes in the intestinal epithelium. HT-29 cells are essentially undifferentiated in culture, but they contain a small proportion (i.e., <5%) of mucus-secreting cells and columnar absorptive cells, a property which offers them heterogeneity [23]. Our findings in this direction indicate the following aspects: (i) the tested commercial EOs are rich in natural compounds, among which estragole (in Hr_EO and Ob_EO), alpha- and beta-citral (in Cc_EO) are present in the highest amount, and (ii) after 48 h of treatment Hr_EO, Ob_EO, and Cc_EO induce cytotoxic effects in CRC cells, which are highly dependent on the tested EO. Considering the possible toxicity of EOs, their effect on the morphology of healthy human keratinocytes (HaCaT cell line) was first evaluated. The preliminary results indicated a weak toxic effect at the highest concentration tested (75 µg/mL), reflected by a slight reduction in the cells’ confluence when compared to Caco-2 CRC cells.

In the case of commercial Hr_EO, the GC-MS analysis revealed the presence of 13 compounds (Table 1), among which estragole represented 63.1% from the total oil composition and could be associated with the biological effects exerted by Hr_EO. Estragole is a representative of the alkenylbenzenes class [24], exerting cytoprotective, antioxidant, and anti-inflammatory properties on gastric cells [25], and pro-apoptotic effects on HepG2 hepatocarcinoma cells [24]. In the realm of colorectal cancer, this phytocompound has not been studied so far. Regarding the in vitro anti-CRC activity of Hr_EO, the most significant effect has been noticed at the highest concentration tested (75 µg/mL), when the cellular viability (Caco-2—87.83%; HT-29—86.91%) and the confluence of Caco-2 cells were significantly reduced (Figure 1 and Figure 4). In addition, at this concentration, apoptotic-like nuclear features (fragmentation, membrane blebbing) and an increase in the AI percentages (to 13.83% in Caco-2 cells, and 94.27% in HT-29 cells) when compared to control were detected (Figure 5 and Figure 6). At a lower concentration (50 µg/mL), Hr_EO inhibited the migration of Caco-2 (WHR = 27.61%) and HT-29 (WHR = 11.78%) cells when compared to control (WHR = 40.73%—Caco-2; WHR = 25.20%—HT-29)—Figure 7. In a previous study, Olsson et al. showed that Sea buckthorn extract exerted a high inhibitory effect on the proliferation of HT-29 cells at concentrations of 0.25% and 0.5%, correlating this observation with the synergistic action between carotenoids, vitamin C, and anthocyanins present in the extract [10,26].

Regarding Cc_EO composition (Table 2), the primary phytochemical constituents are alpha- and beta-citral (37.23% and 28.91%, respectively). Citral is a natural compound possessing well-defined anti-tumor properties, especially in the case of breast carcinoma, stomach, and prostate cancers by inducing apoptosis and inhibiting metastasis [27,28,29,30]. Cc_EO exerted a strong in vitro anti-CRC effect at 75 µg/mL, in terms of cellular viability which decreased to 62.69% in Caco-2 cells and 46.58% in HT-29 cells following the 48 h treatment (Figure 2). These results were accompanied by a significant loss in the Caco-2 cells’ confluence (Figure 7), visible nuclear dysmorphology (condensation and fragmentation), and elevated AI (57.85%) in HT-29 cells at 75 µg/mL (Figure 6). When compared to untreated cells (WHR = 25.20%), Cc_EO manifested a strong inhibitory effect on the viability of HT-29 cells at low (5 µg/mL) and high (50 µg/mL) concentrations with WHR values of 19.49% and 3.40%, respectively. Considering the migration of Caco-2 cells, the effect induced by Cc_EO was statistically insignificant (Figure 7). In a recent study, Ruvinov and colleagues analyzed the anticarcinogenic potency of *C. citratus* ethanolic extract on aggressive CRC cell lines (HT-29 and HCT-116), noting its ability to induce apoptosis, as well as enhance the efficacy of FOLFOX CRC-specific treatment while reducing the adverse events [31].

In Ob_EO, a total of 31 phytocompounds were detected by GC-MS, with estragole (45.9%) being the most prominent (Table 3). This EO potently reduced the cell viability at the highest concentration tested (75 µg/mL–53.36% in Caco-2 cells and 80.67% in HT-29 cells), while at lower concentrations all viability percentages were over 100%. In addition, Ob_EO affected the confluence and adherence of Caco-2 cells at 75 µg/mL (Figure 4), caused nuclear fragmentation and chromatin condensation in both cell lines (Figure 5 and Figure 6), and elevated the AI to 25.03% (Caco-2) and 46.66% (HT-29). Ob_EO significantly increased the cells’ migration rate at 5 (HT-29) and 50 µg/mL (Caco-2) (Figure 7). Fitsiou et al. observed a similar anti-proliferative effect induced by *O. basilicum* EO in Caco-2 CRC cells (EC_50_ = 0.071 ± 0.0032 mg/mL) [32].

With a design to determine the potential irritating effect of the three volatile oils evaluated in the present study, the HET-CAM method was chosen, which uses a biological model including the chorioallantoic membrane of the chicken egg. The results obtained showed that none of the three oils exert an irritating effect on the vascular plexus. As proof of the safety profile, the values obtained for the irritation score of the volatile oils were around one. These results are useful for possible future in vivo tests because volatile oils have a low density and a high lipophilia which favor skin penetration [33]. In regards to *H. rhamoides*, an extract obtained from the leaves of this plant has been previously tested on the chorioallantoic membrane, being noted as having an antiangiogenic effect [34]. *C. citratus* essential oil was evaluated for the local irritant effect using a murine model, with the observation that the essential oil did not exert any toxic effect on the skin [35], data that agree with the ones recorded in the current study. As far as we know, these two volatile oils (*H. rhamoides* and *C. citratus*) have not been tested for the irritating effect by employing the HET-CAM method. On the other hand, in a study on the irritating effect of two hydroalcoholic extracts of *O. basilicum*, Faur et al., assessed the toxic potential at the chorioallantoic membrane, concluding that *O. basilicum* has a good biocompatibility and tolerance at this level [36].

In order to analyze the in vivo effect of the essential oils, different studies were performed. In the case of Hr_EO, anti-tumor properties were reported, especially in the case of cervical cancer, melanoma, and sarcoma [37], and Upadhyay et al. highlighted no adverse effects in subjects after the administration of sea buckthorn oil [38]. In addition, Kumar et al. indicated the beneficial effect exerted by *H. ramnodies* oil on cancer treatment and cancer patients’ good health by counteracting many side effects induced by chemo- or radiotherapy, restoring kidney and liver functions, and increasing appetite [39].

Regarding medicinal usefulness of Cc_EO, Bidinotto and colleagues evaluated the beneficial effects of the essential oil as oral treatment on cell proliferation and apoptosis events, and on early development of hyperplastic lesions in the colon, mammary gland, and urinary bladder induced by N-methyl-N-nitrosourea in female BALB/c mice. Results showed that *C. citratus* oral treatment significantly changed the indexes of apoptosis and/or cellular proliferation for the tissues analyzed, especially on mammary tissue [40]. The effect may be due to beta-citral, the major compound found in the sample. Nguyen et al. concluded that *C. citratus* sample was able to significantly reduce the tumor burden in prostate cancer xenograft models when administered orally, while also being well tolerated [41]. Ob_EO, due to a high level of estragole, is capable of increasing glutathione-S-transferase activity by more than 78% in the liver, stomach, and esophagus—to a strength high enough to be considered as protective agents against carcinogenesis [42]. The anti-inflammatory action was confirmed by Rodrigues et al. using acute and chronic in vivo tests as paw edema, peritonitis, and vascular permeability and granulomatous inflammation model. The anti-inflammatory mechanism of action was analyzed by the participation of histamine and arachidonic acid pathways [43].

## 4. Materials and Methods

### 4.1. Reagents

The essential oils (EOs)—Hr_EO, Cc_EO, and Ob_EO, were purchased from SC Hofigal Export Import SA, BIONOVATIV SRL, and Adams Vision SRL, respectively (Bucharest, Romania). Phosphate saline buffer (PBS), trypsin-EDTA solution, dimethyl sulfoxide (DMSO), fetal bovine serum (FBS), penicillin/streptomycin, and MTT reagent were purchased from Sigma Aldrich, Merck KgaA (Darmstadt, Germany). The cell culture media, Eagle’s Minimum Essential Medium (EMEM-ATCC^®^ 30-2003™), and McCoy’s 5A Medium (ATCC^®^ 30-2007™) were purchased from ATCC (American Type Cell Collection, Lomianki, Poland). N-hexane was purchased from Merck (Darmstadt, Germany). All the reagents were of analytical standard purity and were applied according to the manufacturers’ recommendations.

### 4.2. Gas Chromatography-Mass Spectrometry (GC-MS) Analysis

Before GC-MS analysis, the oils were diluted 1:10 (*v*/*v*) with n-hexane (Merck, Darmstadt, Germany, CAS-No:110-54-3). EOs’ chemical characterization was accomplished using the gas-chromatograph installation with mass spectrometer (GS/MS) QP 2010Plus (Shimadzu, Kyoto, Japan) adapted with AT WAX 30 m × 0.32 mm × 1 μm capillary column. Helium was used as a delivery gas at a flow rate of 1 mL/min. The compounds were submitted to the program: 40 °C for 1 min, a rate of 5 °C/min to 210 °C for 5 min. Injector and ion source temperatures were 250 °C and 220 °C, jointly. An injection volume of 1 μL was used at a split ratio of 1:50. The NIST 02 and Wiley 275 libraries spectra databases were utilized to identify the volatile compounds. The linear retention indices (LRI) were determined in relation to a homologous series of n-alkanes (C8–C24) according to Van den Dool and Kratz formula [44].

### 4.3. Cell Culture

The present study was conducted using two human colorectal adenocarcinoma cell lines—Caco-2 (ATCC^®^ HTB 37™) and HT-29 (ATCC^®^ HTB-38^TM^), which were purchased from ATCC (American Type Cell Collection) as frozen vials. Caco-2 cells were cultured in the EMEM Medium supplemented with 20% FCS, while HT-29 cells were cultured in their specific McCoy’s 5A Medium supplemented with 10% FCS, as presented in literature [45]. Both media contained 1% antibiotic mixture (100 U/mL penicillin/ 100 µg/mL streptomycin) to prevent microbial contamination. The cells were grown under standard conditions: 5% CO_2_ and a temperature of 37 °C in a humidified incubator.

### 4.4. Viability Assay

The cell viability was assessed by applying the MTT technique. Briefly, Caco-2 and HT-29 cells were cultured in 96-well plates (10^4^ cells/200 µL/well) and treated with different concentration of Eos diluted in DMSO (5, 10, 25, 50, 75 µg/mL), followed by 48 h of incubation. Following the treatment period with different concentration of EOs, 10 µL/well of 3-(4,5-dimethylthiazol-2-yl)-2,5-diphenyltetrazolium bromide (MTT) solution (5 mg/mL) was added and the plate was incubated for 3 h, the formazan crystals formed were dissolved (30 min in the dark) in 100 µL of solubilization buffer provided by the manufacturer. Finally, the reduced MTT was spectrophotometrically measured at 570 nm, using the Cytation 5 (BioTek Instruments Inc., Winooski, VT, USA) microplate reader. All experiments were performed in triplicate.

### 4.5. Cell Morphology and Confluence Evaluation

To assess the changes induced by commercial oils in terms of morphology and confluence, the Caco-2 cells were microscopically examined under bright field illumination and pictures were taken at 48 h post-treatment with EOs solubilized in DMSO, at 5, 10, 25, 50, and 75 µg/mL using Cytation 1 (BioTek Instruments Inc., Winooski, VT, USA). The pictures were processed using the Gen5 Microplate Data Collection and Analysis Software (BioTek Instruments Inc., Winooski, VT, USA).

### 4.6. Nuclear Morphology Evaluation

To determine the type of cell death induced by volatile oils, the Hoechst method was performed. The applied protocol followed the manufacturer’s instructions. Briefly, cells were cultured at 1 × 10^5^/well in 12-well plates. After reaching a confluence of approximately 90%, the cells were treated with three different concentrations of EO: 5, 25, and 75 μg/mL. After 24 h of treatment, the medium was removed and 100 μL of staining solution was added to each well diluted 1:2000 in PBS. After incubation for 10 min at room temperature and protected from light, the staining solution was removed and washed three times with PBS. The pictures were taken using Cytation 1 (BioTek Instruments Inc., Winooski, VT, USA) and processed using the Gen5 Microplate Data Collection and Analysis Software (BioTek Instruments Inc., Winooski, VT, USA). The apoptotic index was calculated according to a formula described by Xu et al. [46] and used in one of our previous publications [47].

### 4.7. Wound Healing Assay

To identify the capacity of Hr_EO, Cc_EO, and Ob_EO to interfere with the migration of HT-29 and Caco-2 cells, a wound healing assay was accomplished, following the protocol described in the literature [48]. Succinctly, 2 × 10^5^ cells/well were cultured in 12-well plates, after an 80–90% confluence, a vertical line was drawn in the middle of the well using a sterile pipette tip. The rest of the detached cells was eliminated by washing with PBS. Then, the cells were stimulated with two different concentrations of each oil (5 and 50 µg/mL). Photos were taken at the time interval of 0 h and 24 h using the Olympus IX73 inverted microscope provided with DP74 camera (Olympus Corporation), and cell migration widths were carried out using cell Sense Dimension software.

The scratch closure migration rate (%) was calculated using the following formula [48]:

(1) $$Scratch\;closure\;rate\;=\;\frac{\left(A_{t0}\;-\;A_{t}\right)}{A_{t0}}\;\times \;100$$

where: $A_{t0}$—scratch closure at time 0; $A_{t}$—scratch closure at 24 h.

### 4.8. Chorioallantoic Membrane Assay (CAM Assay)

In this study, the chorioallantoic membrane of the chicken egg was used as a biological model to determine the safety profile and irritant potential of three volatile oils: Hr_EO, Cc_EO, and Ob_EO. The eggs were purchased from a local farmer and prepared for study following the steps described below: (i) on the first day of the experiment, the eggs were washed and disinfected, then data were entered on them and placed in the incubator; (ii) on the third day of incubation, perforations were made in the eggshell and a volume of approximately 7 mL of albumen was extracted in order to allow the detachment of the inner shell membrane of the hen’s egg; and (iii) on the fourth day of incubation, a window was cut at the top of the hen’s egg, large enough to allow the blood vessels to be seen. Then, the perforation was covered with adhesive tape, and the eggs were placed in the incubator until the day the experiment began.

### 4.9. Hen’s Egg Chorioallantoic Membrane (HET-CAM) Assay

The potential irritant effect was determined for the three volatile oils used in the highest concentration (75 µg/mL), previously evaluated in vitro in the cell viability test. For a better quantification of the irritating effect, a negative control represented by water and a positive control represented by sodium dodecyl sulfate (SDS) in a concentration of 1% were selected. The samples and both controls were applied to the chorioallantoic membrane in a volume of 600 µL, to cover the entire surface of the membrane, and the effects were uniform throughout the vascular plexus. After application of the samples, the vascular effects (hemorrhage, lysis, and vascular coagulation) were monitored for a period of five minutes using a steromicroscope (Discovery 8 Stereomicroscope, Zeiss, Göttingen, Germany). Before and five minutes after the application of the samples, photographs of the membrane were taken using Axio CAM 105 color, Zeiss, and these were then processed using ImageJ v 1.50e program (US National Institutes of Health, Bethesda, MD, USA). Finally, the irritating effect was quantified by applying the mathematical formula previously described by Batista-Duharte [49] and applied in our previous studies [50].

(2) $$IS=5\times \frac{301-H}{300}+7\times \frac{301-L}{300}+9\times \frac{301-C}{300}$$

The value of the irritation score provides a classification of the substances, as follows: (i) non-irritating substances (IS = 0–0.9); (ii) irritating substances (IS-1-8.9); and (iii) strongly irritating substances (IS = 9–21) [51].

### 4.10. Statistical Analysis

The experimental data are presented as means ± SD. The differences between data were compared by performing the one-way ANOVA analysis and Dunett’s multiple comparisons post-test. The used software was GraphPad Prism version 9.0.0 for Windows (GraphPad Software, San Diego, CA, USA, www.graphpad.com, accessed on 10 November 2021). The statistically significant differences between data were labeled with * (* *p* < 0.1; ** *p* < 0.01; *** *p* < 0.001; **** *p* < 0.0001).

## 5. Conclusions

The main objective of the current study was to investigate the chemical composition and in vitro efficiency in human colorectal adenocarcinoma cells of essential oils derived from *H. rhamnodies* L., *C. citratus* (D.C.) Stapf, and *O. basilicum* L. species. The data indicate the presence of active phytocompounds (estragole the main compound identified in Hr_EO and Ob_EO, alpha and beta-citral the main compounds from Cc_EO) with a potent EO-dependent in vitro anti-CRC activity at high concentrations. In summary, all EOs decreased the viability and reduced the confluence; Hr_EO and Cc_EO exerted the most potent antimigratory effect, while all samples induced apoptotic specific nuclear features in CRC cells. Furthermore, the results obtained indicate that these essential oils have a relatively low impact on the chorioallantoic membrane, which indicates that these substances exhibit a high biosafety and tolerance profile in the vascular plexus. Further studies, also on other intestinal cells, will be performed to simultaneously examine cell-specific markers and apoptosis indicators and to deepen the mechanisms involved in the anti-tumor efficacy of the studied EOs.

## Figures and Tables

**Table 1:** The main phytochemical constituents of *Hippophae rhamnoides* L. essential oil identified by GC-MS analysis.

Name	R. Time	m/z	Area	Height	Concentration
Isobutvrvl bromide	20.809	TIC	85,682	6956	11.22
Estragole	23.661	TIC	481,549	59,917	63.06
2-Propenoie acid, 2-methyl-, ethenyl ester	25.158	TIC	20,112	3487	2.63
Glycidol	29.778	TIC	18,940	1797	2.48
(S)-2-Hydroxypropanoic acid	30.276	TIC	2732	281	0.35
N-Methylglycine	30.764	TIC	13,019	897	1.70
Propanedioic acid, oxo-, dimethyl ester	31.172	TIC	8017	1029	1.05
2-Methoxy-1, 3-dioxolane	32.226	TIC	13,747	813	1.80
Propane, 2-ethoxy	32.600	TIC	1895	404	0.24
Isopropyl Alcohol	33.008	TIC	10,255	784	1.34
Ethanol, 2-[2-(ethnyloxy) ethoxy]-	33.162	TIC	9418	941	1.23
Ethane, 1,1′-oxybis[2-methoxy]	34.232	TIC	2883	831	2.99
Pentandioic acid, (p-t-butylphenyl) ester	34.553	TIC	15,391	12,088	9.87

**Table 2:** The main phytochemical constituents of *Cymbopogon citratus* (D.C.) Stapf essential oil identified by GC-MS analysis.

Name	R. Time	m/z	Area	Height	Concentration
.apha, -Thujene	6.006	TIC	64,743	10,639	0.072
Bicyclo [3.1.0]hex -2-ene, 2-methyl-5-(methy)	6.466	TIC	44,0792	41891	0.487
Camphene	7.511	TIC	1,129,013	136,064	1.247
.beta. –Myrcene	10.179	TIC	1273,812	163,422	1.407
Limonene	11.283	TIC	2693,725	336,122	2.976
Ocimene (E)-	12.219	TIC	284,397	45,290	0.314
Ocimene (Z)-	12.712	TIC	164,002	27,220	0.181
4 –Nonanone	14.808	TIC	873,286	143,765	0.965
5-Hepten-2-one, 6-methyl-	14.972	TIC	2,307,111	355,154	2.549
Phenol, 3-methyl-5-(1-methylethyl)-, Methylca	16.779	TIC	34,226	8564	0.038
2-Propenoic acid, 2-methyl-, ethenyl ester	17.253	TIC	40,657	7065	0.045
1,6-Heptadiene, 2-methyl-	18.953	TIC	192,620	27,349	0.213
d -Norbomanone	19.136	TIC	100,006	17,289	0.110
Copaene	19.610	TIC	61072	10,909	0.067
2,2-Dimethylocta-3, 4-dienal	19.757	TIC	265,918	29,040	0.294
Linalool	20.796	TIC	2,900,499	181,609	3.204
Cyclopropane, 1,1-dimethyl-2-(2-methyl-2-pro)	21.310	TIC	798,964	80,284	0.883
Cyclopentanecarboxylic acid, 2 methyl-3-meth	21.991	TIC	191,346	35,596	0.211
1,7 -Octadiene, 3-methylene-	22.149	TIC	76,920	13,393	0.085
Caryophyllene	22.483	TIC	2,021,644	265,029	2.233
Estragole	23.676	TIC	231,331	26,376	0.256
beta Citral (Z)-	24.025	TIC	26,167,607	3,452,865	28.910
n-menth-1-en-8-ol	24.953	TIC	808,078	74,951	0.893
alpha Citral (E)-	25.203	TIC	33,701,032	4,346,928	37.233
Geraninol acetate, (Z)-	25.698	TIC	5,267,532	966,974	5.820
delta Cadinene	26.157	TIC	106,187	21,685	0.117
gamma Muurolene	26.286	TIC	722,205	113,718	0.798
Caryophyllene oxide	31.129	TIC	180,807	32,715	0.200
3-Buten-2-ol, 2,3-dimethyl-	31.600	TIC	97,200	22,430	0.107
Geraniol cis(Z)-	32.427	TIC	847,203	40,371	0.936
trans Geraniol (E)-	33.245	TIC	6,074,456	249,366	6.711
Geranic acid	37.190	TIC	145,179	28,938	0.160
Isoeugenol	37.636	TIC	251,368	51,836	0.278

**Table 3:** The main phytochemical constituents of *Ocimum basilicum* L. essential oil identified by GC-MS analysis.

Name	R. Time	m/z	Area	Height	Concentration
alpha Thujene	6.367	TIC	76,945	12,186	0.086
Beta Myrcene	10.169	TIC	64359	8994	0.072
Cyclobutane, 1,3-diisopropenyl-, trans	11.274	TIC	75,600	11,946	0.084
Pentane, 3-bromo-	11.495	TIC	87,739	12594	0.098
cis Ocimene	12.703	TIC	328,154	51,598	0.365
Oxalic acid, cyclobutyl, ethyl ester	13.722	TIC	32,818	4744	0.037
2,3-Hexanedione	14.383	TIC	27,218	4452	0.030
5-Hepten-2-one, 6-methyl-	14.970	TIC	193,362	35,549	0.215
Linalool oxide	18.840	TIC	142,022	13,853	0.158
beta Linalool	20.748	TIC	32,664,606	2,147,497	36.375
alpha Bergamotene	22.058	TIC	674,455	102,291	0.751
beta Caryophyllene	22.482	TIC	502,819	75,620	0.560
beta Famescene	23.426	TIC	44,046	8090	0.049
Estragole	23.735	TIC	41,294,691	5,420,144	45.985
cis Citral	24.005	TIC	1,938,577	276,407	2.159
.alpha.-Caryophyllene	24.208	TIC	156,947	27,279	0.175
beta Famescene	24.208	TIC	156,947	27,279	0.175
1,6-Octadiene, 2,6-dimethyl-	24.930	TIC	127,610	20,567	0.142
trans Citral	25.171	TIC	1,594,351	276,329	1.775
1,6,10-Dodecatriene, 7,11 dimethyl-3 methyle-	25.389	TIC	36646	9762	0.041
Geranyl isobutyrate	25.680	TIC	61,547	11,094	0.069
cis alpha Bisabolene	26.434	TIC	1,926,980	319,323	2.146
3-Methylbenzothiophene	27.345	TIC	14,437	4604	0.016
Cinnamaldehyde	31.855	TIC	63,862	12,081	0.071
1-Heptyn-4-ol	32.343	TIC	50,217	5030	0.056
Isoeugenol	33.943	TIC	55,670	5993	0.062
Thymol	34.423	TIC	6,085,833	489,592	6.777
Carvacrol	35.287	TIC	1,083,683	96,648	1.207
Benzofuran	39.662	TIC	18,232	3811	0.020
Butane, 1-methoxy-3-methyl-	39.893	TIC	19,062	3160	0.021
3-Methoxycinnamaldehyde	41.306	TIC	200,139	41,695	0.223

**Table 4:** Irritation score values for positive control (sodium dodecyl sulfate 1%), negative control (distilled water), *H. rhamnoides* L., *C. citratus* (D.C.) Stapf and *O. basilicum* L. essential oils (Hr_EO, Cc_EO, and Ob_EO).

	SDS 1%	H_2_O	Hr_EO	Cc_EO	Ob_EO
IS	19.68	0.10	1.12	0.99	1.26
tH	15 s	300	300	300	300
tL	18 s	300	273	295	268
tC	24 s	299	286	273	285

SDS—sodium dodecyl sulfate; IS—irritation score; tH—time of hemorrhage; tL—time of lysis; tC—time of coagulation.

## Data Availability

Data presented in this study are available on request from the first author.
